# Therapeutic Monoclonal Antibody Therapies in Chronic Autoimmune Demyelinating Neuropathies

**DOI:** 10.1007/s13311-022-01222-x

**Published:** 2022-03-28

**Authors:** Chiara Briani, Andrea Visentin

**Affiliations:** 1 Department of Neurosciences, Neurology Unit, University of Padova, Padova, Italy; 2 Department of Medicine, Hematology Unit, University of Padova, Padova, Italy

**Keywords:** Chronic Inflammatory Demyelinating Polyradiculoneuropathy (CIDP), Multifocal Motor Neuropathy (MMN), anti-MAG antibody neuropathy, Rituximab, Obinutuzumab, Eculizumab, Nodopathies, Complement, Neonatal Fc receptor, Fc receptor

## Abstract

Autoimmune diseases of the peripheral nervous system have so far been treated mainly with exogenous high-dose intravenous immunoglobulins (IVIg), that act through several mechanisms, including neutralization of pathogenic autoantibodies, modulation of lymphocyte activity, interference with antigen presentation, and interaction with Fc receptors, cytokines, and the complement system. Other therapeutic strategies have recently been developed, in part to address the increasing shortage of IVIg, prime among which is the use of B cell depleting monoclonal antibodies, or small molecule inhibitors targeting the B-cell specific kinases. Rituximab, a chimeric monoclonal antibody against CD20 + B lymphocytes, is currently the most used, especially in anti-MAG antibody neuropathy and autoimmune neuropathies with antibodies to nodal/paranodal antigens that are unresponsive to IVIg. After several reports of its efficacy in chronic inflammatory demyelinating polyradiculoneuropathy (CIDP), rituximab is currently under investigation in three Phase 2 trials in CIDP. In addition, the possible role of complement activation in the pathogenesis of chronic autoimmune neuropathies has brought into consideration drugs that can block the complement cascade, such as eculizumab, a monoclonal antibody already assessed in acute polyradiculoneuropathies, and approved for myasthenia gravis. Preliminary data on eculizumab in multifocal motor neuropathy have been published, but randomized controlled studies are pending. Moreover, the neonatal Fc receptor, that recycles IgGs by preventing their lysosome degradation, is an important and attractive pharmacological target. Antibodies against FcRn, which reduce circulating IgG (both pathogenic and non-pathogenic) have been developed. The FcRn blocker efgartigimod, a humanized IgG1-derived Fc fragment, which competitively inhibits the FcRn, has recently been approved for the treatment of myasthenia gravis and is currently under investigation in CIDP. In addition, the anti-human FcRn monoclonal antibody rozanolixizumab is currently being assessed in phase 2 trials in CIDP. However, none of the abovementioned monoclonal antibodies is currently approved for treatment of any immune-mediated neuropathies. While more specific and individualized therapies are being developed, the possibility of combined treatments targeting different pathogenic mechanisms deserves consideration as well.

**Supplementary Information:**

The online version contains supplementary material available at 10.1007/s13311-022-01222-x.

## Introduction

Monoclonal antibodies have recently gained interest in the treatment of immune-mediated neuropathies, particularly when there is evidence of underlying humoral pathogenetic mechanisms.

More data are available for the polyneuropathy with antibodies to myelin-associated glycoprotein (MAG), but increasing evidence is also emerging for other immune-mediated diseases of the peripheral nervous system, including **c**hronic inflammatory demyelinating polyradiculoneuropathy (CIDP) and autoimmune neuropathies with antibodies to nodal and paranodal antigens.

Moreover, a potential pathogenic role of complement in chronic autoimmune neuropathies [1] may open new therapeutic avenues with drugs inhibiting complement activation. Eculizumab, a recombinant humanized monoclonal antibody that binds and sequesters C5a, prevents its enzymatic cleavage by the C5 convertase into C5a and C5b, thus inhibiting C5b-9 membrane attack complex (MAC) formation. Eculizumab has already been approved in myasthenia gravis and is under investigation in acute polyradiculoneuropathies [2–5].

A further potential therapeutic target in immune-mediated polyneuropathies is the neonatal Fc receptor (FcRn), known to facilitate IgG recycling and protection from degradation, thereby extending the half-life of IgG molecules [6]. High-dose intravenous immunoglobulins (IVIg), currently used in several immune-mediated diseases, act through several mechanisms, including competition with pathogenic autoantibodies for FcRn binding, saturating the receptor and thus increasing IgGs turnover [7]. Monoclonal antibodies against FcRn may be effective in reducing serum levels of pathogenic IgG autoantibodies without removing other circulating factors. The FcRn blocker efgartigimod has recently been approved by the U.S. Food and Drug Administration for the treatment of anti-acetylcholine receptor antibody positive myasthenia gravis and is currently under investigation in CIDP.

However, even if they hold promise, none of the above-mentioned therapeutic monoclonal antibodies are currently approved for treatment of any of the immune-mediated neuropathies. In the present paper we’ll report on the currently used monoclonal antibodies in the treatment of chronic immune-mediated neuropathies, and present preliminary data on new potential therapeutic strategies.

## Chronic Inflammatory Demyelinating Polyradiculoneuropathy

Chronic inflammatory demyelinating polyradiculoneuropathy (CIDP) is an inflammatory polyradiculoneuropathy characterized by progressive (more than 2 months) symmetric or relapsing–remitting sensory-motor deficits. Recently the CIDP criteria have been revised, and the chronic inflammatory sensory polyradiculopathy (CISP) and the autoimmune neuropathies with antibodies to nodal-paranodal antigens (neurofascin, contactin, contactin-associated protein 1-(caspr1)) are no longer classified as CIDP [8].

There are no pathognomonic clinical or biochemical markers of CIDP, and the diagnosis is based on a combination of clinical, electrophysiological and supportive criteria [8].

Randomized controlled trials have shown efficacy of steroids, plasma exchange, and IVIg for the treatment of CIDP with up to 70% of patients responding to each of these treatments [9]. The updated European Academy of Neurology/Peripheral Nerve Society CIDP guidelines affirm the use IVIg or corticosteroids in typical CIDP and CIDP variants in the presence of disabling symptoms, while plasma exchange is considered similarly effective but less well tolerated [8]. Subcutaneous Ig is strongly recommended for maintenance treatment [8, 10]. In CIDP patients who are not responsive or become refractory to first line therapies, immunosuppressants have been used [11] despite the lack of evidence of efficacy in the few performed controlled studies [12]. Among the alternative therapies, rituximab seems to be the most promising. The first patient with CIDP responsive to rituximab after failure of IVIg and steroids was reported in 2004 [13]. The patients had a concurrent IgM/k low-grade-small-B-cell lymphoma, with no anti-MAG antibodies. Rituximab (375 mg/m^2^ for 4 weeks) was administered with progressive dramatic improvement. Several case reports and small case series followed [14, 15] suggesting that CIDP, especially when associated with other autoimmune or hematological diseases responds to rituximab in a percentage ranging from 69 to 75% [14, 15]. Rituximab appears to also be effective in a retrospective study on patients with refractory CIDP [16]. A recent systematic review and a meta-analysis of rituximab treatment in CIDP patients (including patients with IgG4 antibodies to nodal or paranodal antigens) estimated an efficacy around 75% [17]. Currently, two Italian [9] and one Japanese [18] clinical trials are ongoing to determine the efficacy and safety of rituximab in patients with CIDP responsive to IVIg and in patients with refractory CIDP.

Pathological data from nerves of patients with CIDP point to a possible role of complement activation [1]. It is not known whether eculizumab may be effective in CIDP, either as single agent or as add-on therapy. Data are available only for the rare genetic forms of relapsing–remitting polyneuropathies in patients lacking the homozygosous p.Cys89Tyr mutation on CD59 [19]*.* Since the CD59 protein inhibits the final step of MAC, the affected patients are susceptible to hemolysis, cerebrovascular events, and severe chronic relapsing–remitting polyneuropathy [20, 21]. An open 2-year treatment with eculizumab in these patients has been shown to improve chronic hemolysis and prevent neurological worsening, in conjunction with neurophysiological improvement in a few cases [19]. 

The neonatal Fc receptor (FcRn), that favors IgG recycling and extends the half-life of IgG molecules, including pathogenic IgG autoantibodies [6], has become a potential therapeutic target in neurological autoimmune diseases. Antibodies against the FcRn, also called Abdegs (antibodies that enhance IgG degradation) [22] have been found to be effective at reducing levels of circulating IgG (both pathogenic and non-pathogenic), with the added advantage—distinct from plasma-exchange—of not removing other circulating factors (e.g. albumin, other isotypes of antibodies, and clotting factors) or interfering with the complement pathway and other immune cells.

The FcRn blocker efgartigimod, a humanized IgG1-derived Fc fragment which competitively inhibits the FcRn, has recently been approved by the U.S. Food and Drug Administration for the treatment of anti-acetylcholine receptor antibody-positive myasthenia gravis, and is currently under investigation in CIDP (Table 1). Also the subcutaneous rozanolixizumab is currently being investigated in CIDP (Table 1).

Safety concerns regarding the use of Abdegs in treating autoimmune neurological diseases are mainly related to IgG depletion [23, 24].

Finally, the receptors for the Fc portion of immunoglobulins play a pivotal role in humoral and innate immunological homeostasis, are responsible for effector functions and are involved in several autoimmune diseases [25]. Recent experimental data point to the role of Fc-gamma receptors in animal model of inflammatory neuropathies [26, 27]. Consistently, the inhibition of Fcγ receptors may become a target of autoimmune diseases [26, 28, 29]. With the help of Fc engineering techniques [30], several selective therapeutic option are currently under development and investigation not only for immune-mediated diseases but also for infections and cancer treatment [31].

## Nodo-paranodopathies

Nodo- and paranodopathies are a group of recently described autoimmune neuropathies, currently excluded from the CIDP classification [8]. These patients have peculiar phenotypic features, are generally younger than in classical CIDP, and have poor response to IVIg and antibodies—mainly of IgG4- and IgG3-subtype—to nodal-paranodal antigens namely neurofascin-155, contactin-1 and caspr1 in the paranodal region, and neurofascin-186/-140 in the nodal region [32–35]*.*

The diseases with anti-neurofascin antibodies present as aggressive, predominantly distal sensory-motor neuropathy associated with sensory ataxia and disabling tremor, where the form with anti-contactin antibodies may have a motor predominance and early axonal damage [36].

Rarely patients may present with very aggressive, life-threatening neuropathy associated with pan-neurofascin antibodies [37, 38]*.*

Nodopathies with antibodies to caspr1/contactin-1 complex have also been described. These patients present with rapidly progressive and disabling neuropathies, with pain in half of the cases and cranial nerves involvement (ophthalmoparesis, facial or oropharyngeal weakness) in 40%, as well as poor response to IVIg therapy [39].

These neuropathies differ from the classical CIDP not only for the peculiar clinical features [40] and little response to IVIg, but also for the lack of inflammation or macrophage-mediated demyelination at neuropathology [41–43]*.* The poor response to IVIg is likely due to the common IgG4 isotype of the antibodies, which are unable to bind the first C1q complement component so failing to activate the complement cascade [44]. These autoimmune neuropathies seem instead to respond to rituximab [33, 34, 45]. Recently a single 52-year-old patient affected by a severe neuropathy with antibodies to neurofascin and concomitant late onset nemaline myopathy as well as smoldering multiple myeloma, was treated with daratumumab, the humanized anti-CD38 monoclonal antibody targeting long-lived plasma cells, after failure of several therapies, including steroids, plasma-exchange and rituximab [46]. In the reported case, besides clinical improvement (INCAT disability scale changed from 7 to 0), also antibody titer decreased. Caution is however needed, considering that in the heterogeneous small cohort of 7 refractory patients with antibody-mediated neurological diseases, severe side effects related to daratumumab therapy occurred in 5 patients, including one death.

## Multifocal Motor Neuropathy

Multifocal motor neuropathy (MMN) is a rare, acquired, motor neuropathy characterized by progressive asymmetric weakness with no sensory loss. The disease is slowly progressive it affects mainly young men with common onset al distal upper limbs, and electrophysiologically is characterized by the presence of conduction blocks [47]*.* Antibodies against the monosialoganglioside GM1 are present in up to 60% of the patients and the dosage sensitivity increases when galactocerebroside is associated in the ELISA assay [48]*.*

The pathogenic mechanism of MMN is still not clearly understood, but the possible role of complement in the pathomechanism is suggested by experimental data [49–52] and by the response to IVIg therapy [53, 54]. The disease is slowly progressive and patients will require over time increasing IVIg dosage. Subcutaneous Ig are also used in maintenance therapy [54, 55].

IVIg may however lose efficacy, and also the guidelines suggest to consider immunosuppressive treatment if IVIg is not enough, but unfortunately good alternative therapies are still lacking.

MMN is not responsive to steroids or plasma exchange, and their use is not recommended [53]*.* No immunosuppressant drugs have been proven efficacious in controlled trials [56]. Rituximab has been used with the aim to reduce the IVIg dosage or to stabilize the progression of the disease [57–60] but no randomized controlled trials have so far been performed.

Considering the role of complement in the pathogenesis of MMN, eculizumab has also been considered as potential therapeutic strategy. In 2011 an open-label study investigated eculizumab in 13 MMN patients, ten of whom were concurrently treated with IVIg, has been performed [61]*.* Preliminary data are promising as safety of the drug (safety and tolerability of eculizumab in patients receiving IVIg were the primary aims of the study), but the benefit was only marginal, with no objective (MRC sum score; pinch/palm grip using dynamometer, 9-hole peg test, 10-m walk time, self-evaluated functional rating scale, Overall neuropathy limitation scale, ONLS; European quality of life scale) measurable improvements. No severe adverse events occurred, and no worsening of MMN was reported. Moreover, the majority of patients needed continuous IVIg therapy while in eculizumab treatment, suggesting that IVIg benefit may be independent of complement activation. Longer and controlled trials are warranted.

A possible use of ARGX-117, a humanized, Fc-engineered human IgG1 inhibitory anti-C2 antibody, is suggested by promising experimental data [52]. In a MMN model, Budding et al. et al. investigated complement activation by anti-GM1 IgM antibodies from MMN patients sera applied to motor neurons derived from induced pluripotent stem cells. The authors found that IgM anti-GM1 binding to motor neurons triggers complement activation, which is C2-dependent, and is inhibited by ARGX-117, an antibody targeting C2, that may be therefore a potential therapeutic target for MMN.

## Anti-MAG Antibody Neuropathy

Neuropathy with anti-MAG antibodies is the most common IgM paraproteinemic neuropathy, characterized by predominant sensory symptoms, ataxic gait, tremor at upper limbs, with motor involvement and disability occurring late in the course of the disease [62]*.* Despite slowly progressive, the neuropathy may severely affect functional activities and quality of life [63–65].

The IgM paraprotein is commonly a monoclonal gammopathy of undetermined significance (MGUS), but it may underscore also a lymphoproliferative disorder, most commonly Waldenstrom’s Macroglobulinemia (WM) but also marginal zone lymphoma or chronic lymphocytic leukemia (CLL). In these cases, the choice of treatment generally depends on the severity of the neuropathy, within other parameters of the underlying non-Hodgkin lymphoma.

No adequate immunotherapy has so far been shown to be effective in anti-MAG neuropathy [66]*.* After promising results from small uncontrolled studies, rituximab has been assessed in two randomized controlled trials [67, 68], accounting only for MGUS patients, WM being an exclusion criterium, with controversial results. The first study enrolled 26 patients undergoing a single course of rituximab (at a standard dose of four infusions of 375 mg/m^2^ every week for four weeks) or placebo [67]. In the 13 patients treated with rituximab, 4/13 improved by ≥ 1 point on the Inflammatory Neuropathy Cause and Treatment (INCAT) disability leg score, and the majority of them (69%) showed significant improvement in the ‘time to walk 10 m’. The second study included 54 patients treated with a single course of rituximab or placebo [68]. Changes were observed in the rituximab group only in some secondary outcome measures, including improvement by ≥ 2 points on the INCAT disability scale, improvement in the self-evaluation scale and in two sub-scores of the Short Form 36 questionnaire. A comparison between the two studies is difficult, because of the different inclusion criteria and the different disease duration before starting treatment (12.9 yrs in the first study vs 3.8 in the second study, respectively). The failure of the two controlled studies to detect clinically meaningful changes may be related to the lack of proper measures sensitive to changes in a neuropathy that, at least in the first period, is mainly sensitive.

The last Cochrane meta-analysis [66]*,* including the two trials, showed that rituximab was actually effective in improving disability scales, especially INCAT [69], and in the response to questionnaires of the global impression of the disease. Regarding adverse effects, rituximab was well tolerated with mild infusion-related reactions such as nausea, fever, headache, hypotension, lightheadedness, and erythematous rash with itching being the most common side effects. Despite the two controlled trials enrolled only patients with IgM MGUS (being WM an exclusion criterion), patients with anti-MAG antibody neuropathy and WM display a similar response to rituximab, with a more persistent benefit in patients with short disease duration [70]*.*

No factors (biological or immunological) have so far been identified as being associated with response to rituximab [71–73]*.* Moreover, worsening of the neuropathy has also been reported [74–77] even if the exact mechanism is not well defined. In WM the sudden death of neoplastic clone can lead to the release of the paraprotein in the blood, with acute increase of the IgM monoclonal component and of blood hyperviscosity, sometime causing a hyperviscosity syndrome. This complication is known as “IgM flare” [78]. It is likely that some worsening of neuropathic symptoms after rituximab treatment might be related to the release of anti-MAG antibodies from dying B-cells, an “IgM antiMAG flare” [76, 79] or may be due to cytokine release [80].

Additional potential therapies might include rituximab with associated chemotherapeutic agents, like chlorambucil or bendamustine [81]. Despite the efficacy of these combinations to improve the patients’ symptoms and to decrease IgM levels, they are associated with increased hematological cytopenia and potential long-term toxicities, like infections and secondary cancers, if compared with rituximab single-agent and other novel drugs (see afterward) [82–85].

New, more effective in B cells depletion, monoclonal antibody might be a potential alternative to rituximab.

**Fig.1:**
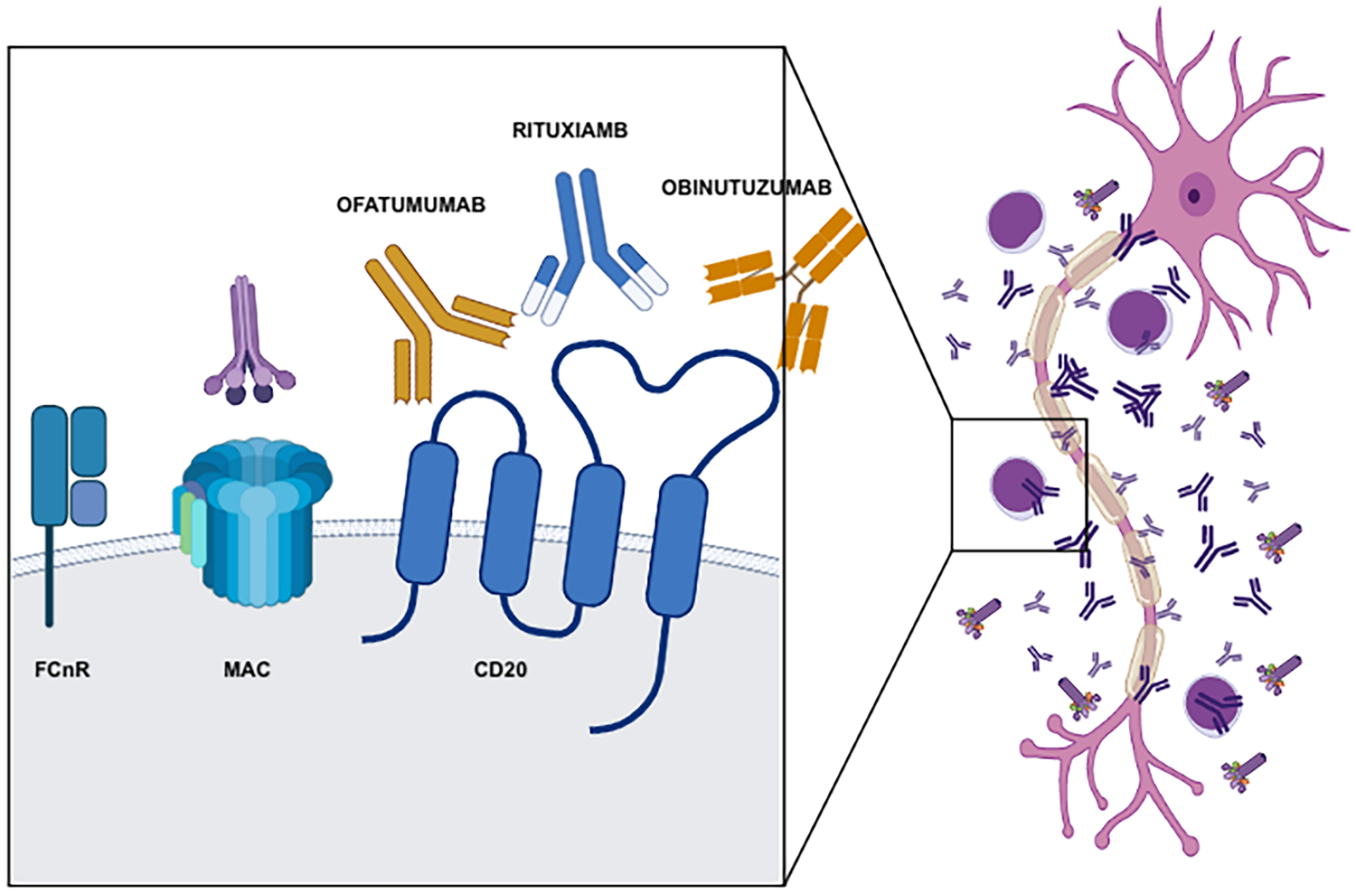
In the right panel of the figure, there is a representative neuron surrounded with lymphocytes, either B or T cells, antibodies, and complement. In the left panel of the figure, there is a magnification of B lymphocyte membrane showing neonatal Fc receptor, complement membrane attack complex (MAC), CD20, and monoclonal antibodies targeting CD20. Rituximab is a chimeric anti-CD20 antibody with both murine and human fragments. Ofatumumab is a fully humanized anti-CD20 antibody which binds CD20 at a site different from rituximab. Obinutuzumab is a fully humanized glycol-engineered anti-CD20 antibody, made to enhance receptor binding

Ofatumumab is a fully humanized anti-CD20 monoclonal antibodies which bind CD20 to a different site than rituximab (Fig. 1). Despite its initial promising activity, also in rituximab-refractory hematological patients, and lower rate of IgM flare, this drug is no longer used in hematology due to the advent of more effective drugs [78, 86]. However, subcutaneous ofatumumab has been recently approved by the U.S. Food and Drug Administration and the European Medicines Agency (EMA) for the treatment of patients with multiple sclerosis [87].

Obinutuzumab, previously known as GA101, is a novel, type II, glycoengineered, humanized anti-CD20 monoclonal antibody developed to address the need for novel therapeutics with higher activity than rituximab (Fig. 1). The post-translational glycoengineering process used in the development of this agent enhances its binding affinity to the FcγRIII receptor on immune effector cells [88, 89]. The structure and the glyco-engeneering of obinutuzumab act enhancing direct cell death, antibody-dependent cyto-toxicity and cellular phagocytosis, while decreasing complement-dependent cytotoxicity. Rituximab, by comparison, works primarily via complement-dependent cytotoxicity (by clustering CD20 within lipid rafts) and by antibody mediated cyto-toxicity and cellular phagocytosis, with direct cell death contributing much less to the overall antitumor activity [90]. Mossner et al. showed obinutuzumab to be 10–25 times more potent and 1.5–2.5 times more effective than rituximab in depleting B-cells in whole blood from healthy human donors (p < 0.001) [89]. Obinutuzumab has occasionally been used in anti-MAG antibody neuropathy. Rakocevic et al. used obinutuzumab to treat two patients with IgM gammopathy and anti-MAG antibody neuropathy who were unresponsive to rituximab. Despite hematological response (decline in IgM and anti-MAG antibodies levels) no clinical improvement was reported, possibly due to long disease duration and irreversible axonal nerve damage [91]. Subsequently Briani et al. reported on two drug-naive patients with anti-MAG antibody neuropathy and CLL who were treated with obinutuzumab and chlorambucil as first-line therapy, with significant clinical and neurophysiological improvement. However, both patients required hospitalization for severe pneumonia [92]*.*

In recent years, mutational screenings in patients with monoclonal gammopathies have identified recurrent somatic mutations of the MYD88 gene. In particular, the MYD88 ^L265P^ mutation was the most common in patients with WM and with less extent in patients with IgM MGUS. The constitutive activation of MYD88 activates the downstream pathway, such as Bruton’s tyrosine kinase (BTK) and NF-kB proteins, which favor cancer cell survival and proliferation [93]. The identification of this mutation might become clinically relevant in the diagnosis and therapy of patients with IgM paraproteinemic neuropathy. In addition, acquired mutations in the C-terminal domain of CXCR4 gene have been reported in WM and shown to be associated with a more aggressive disease. More importantly, MYD88/CXCR4 status has been shown to be predictive of the response to ibrutinib in WM [94]*.* Specifically, WM patients with MYD88 mutation and wild type CXCR4 have been shown to have better and longer responses to ibrutinib. Among the 63 patients studied by Treon et al. [94], nine—three with anti-MAG antibodies—had received ibrutinib for progressive IgM paraproteinemic neuropathy. All nine patients achieved a hematological response. Subjective improvements of neuropathy occurred in 5 patients and remained stable in 4 patients during the treatment course. In a subsequent study [95] four of 31 rituximab refractory WM patients were treated with ibrutinib for the neuropathy: two remained stable and two had subjective improvement starting from week 9 of treatment, with subsequent complete recovery in one patient. Although the hematological evaluation might have lacked specific (clinical or neurophysiological) neurological scales to properly grade the neuropathic response to ibrutinib, these preliminary data are promising and indicate that ibrutinib does not worsen neuropathy and may improve it, thus offering a potential therapeutic option in IgM paraproteinemic polyneuropathies [93, 96]. Recently we reported on the first 3 patients with anti-MAG antibody neuropathy and WM (MYD88^L265P^ mutation and wild-type CXCR4 gene), treated with ibrutinib [97]. Two of them had previously been treated with rituximab with progressive loss of efficacy. The oral drug ibrutinib was well tolerated, and no atrial fibrillation nor infections occurred. In addition, second generation BTK inhibitors, such as acalabrutinib [98] and zanubrutinib [99] are under clinical investigation as single agent or in combination with anti-CD20 monoclonal antibodies for the treatment of symptomatic WM. These new drugs are highly selective on BTK and are associated with lower adverse events than ibrutinib [100]. The latter also seems to be active in MYD88 wild-type patients [101].

Venetoclax is an oral and selective BCL2 inhibitor that in combination rituximab has proven to be highly effective in B-cell malignancies even after ibrutinib failure [102]. Recently, Castillo et al. showed that venetoclax was able to induce remission regardless of CXCR4 mutations [103].

Although pathological data demonstrate that the damage to myelin in anti-MAG neuropathy is complement-mediated, [104, 105], no complement fractions (C3b, C3bi and C3c) have been found in the serum from a subgroup of therapy-naive patients with anti-MAG antibody neuropathy [106].

## Concluding Remarks

In conclusion, among the currently available monoclonal antibodies, rituximab is the most used in chronic immune-mediated neuropathies. Despite being effective in less than half of patients with anti-MAG antibody polyneuropathy, in clinical practice it remains the treatment of choice, thanks to its favorable safety and tolerability profile. Occasional reports on the more active and B cell-efficacious obinutuzumab need to be confirmed and weighted against side effects, which require caution especially in older patients.

Rituximab is also the main therapeutic choice in autoimmune neuropathies with IgG4 antibodies to nodal and paranodal antigens [45], where the IVIg fails to give benefit likely due to the inability of the antibodies to activate the complement pathway. Maintenance therapy is empirically proposed based either on clinical response/relapse [44] or on immunologic data (antibody titer or memory B-cell monitoring). Hopefully, the results of the two Italian [9] and one Japanese [18] clinical trials currently ongoing in patients with refractory CIDP or CIDP responsive to IVIg will help determine the efficacy and safety of rituximab in CIDP. Whether rituximab will require maintenance therapy, as in some hematological diseases and in some cases of autoimmune nodopathies, needs to be investigated.

Preliminary positive data on the BTK inhibitor, ibrutinib, in patients with anti-MAG antibody neuropathy and a specific mutational profile (MYD88^L265P^ mutation, wild-type CXCR4 gene) need further confirmation on larger populations. The BTK inhibitors (ibrutinib, zanubrutinib, rilzabrutinib) have a potential therapeutic role in B-cell-mediated diseases other than anti-MAG antibody neuropathy, given their good profile (oral administration and favorable safety profile). A phase 2 trial with acalabrutinib and rituximab is currently ongoing (Table 1). Whether these oral drugs prove to be beneficial in combination with anti-CD20 monoclonal antibodies is unknown and deserves investigation through multicenter randomized clinical trials.

On the contrary, MYD88 wild-type and/or the rare CXCR4-mutated patients might benefit from second generation BTK inhibitors or other agents like venetoclax (personal experience).

With regard to complement-targeted therapies, the efficacy of eculizumab in patients lacking the CD59 protein for homozygous p.Cys89Tyr suggests that the antibody might be efficacious, at least as add on therapy, as well as in other conditions where the complement pathway is altered. Currently, studies with eculizumab and with C1q inhibitors are being performed in Guillain-Barré syndrome, whereas in CIDP a Phase 2 trial with Cs1 inhibitor is ongoing (Table 1). Preliminary open data with eculizumab in MMN patients failed to show advantage or possibility of reducing IVIg dosing, but larger studies, hopefully including therapy-naive patients, might be considered.

Trials with eculizumab on paroxysmal nocturnal hemoglobinuria, myasthenia gravis, and neuromyelitis optic spectrum disorders showed good safety results, but caution is needed for the risk of meningococcal infections (requiring prophylactic vaccination against certain encapsulated bacteria) as reported in Guillain–Barre Syndrome studies [107].

The selectivity of the FcRn blockers seems safe and creates many expectations in the treatment of neurological diseases. After the approval of efgartigimod, the first recombinant antibody-based therapy for selective IgG depletion in myasthenia gravis, the scientific community is eager to know the results from the ongoing trials in CIDP with efgartigimod and rozanolixizumab.

The possibility of combined therapies acting on different, non-competing, targets should be considered, e.g. the use of FcRn blockers or IVIg at a dosage capable of saturating the FcRn, together with agents acting on the complement pathway, at least in diseases where IVIg is efficacious and there is evidence of a pathogenic role for complement. Next generation antibody-based therapies [108], and next generation complement therapies [109] are a growing field and a challenging and enthusiastic road lies ahead as well in the context of neurological diseases, especially considering the increasing shortage of IVIg that requires alternative and more tailored therapies.

Interesting potential targets, susceptible to selective therapy, are also the Fcγ receptor, and monoclonal antibodies against both the activating Fcγ receptors or the inhibitory FcγIIB have been developed and already assessed in the oncological setting [110].

Limiting factors are the rarity of the chronic immune-mediated polyneuropathies and the associated difficulties to carry out multicenter studies with proper and validated outcome measures and adequate biochemical biomarkers. Alternative trials, such as platform designed trials or trials within cohorts (TwiCs) might be taken into consideration to optimize resources and limit costs.

**Table 1:** Active and ongoing clinical trials in chronic immune-mediated neuropathies

TRIAL NUMBER	PHASE	DRUGS	TARGETS
Anti-MAG antibody neuropathy			
NCT04568174	phase 1	PPSGG	MAG antibody
NCT03701711	Phase 1	Lenalidomide	Cereblon
NCT05065554	Phase 2	Acalabrutinib-rituximab	BTK + CD20
NCT05136976	Phase 3	Rituximab	CD20
NCT00050245			
Chronic inflammatory demyelinating polyneuropathy			
NCT04281472	Phase 2	Efgartigimod	FcRn
NCT03861481	Phase 2	Rozanolixizumab	FcRn
NCT03864185	Phase 2	Rituximab	CD20
NCT04480450			
NCT01236456	Phase 2	Cyclophosphamide	DNA
NCT00278629	Phase 2	Cyclophosphamide + anti-thymocyte globulin	DNA + CD3
NCT04658472	Phase 2	SAR 445,088	C1s
NCT00099489	Phase 2	Interferon Beta-1a	Interferon Beta-1a
NCT01625182	Phase 2	Fingolimod	
NCT01184846	Phase 3	Immunoglobulin ev Privigen	
NCT01824251	Phase 3	Immunoglobulin ev NPB-01	
NCT03166527	Phase 3	Immunoglobulin ev Pazynga	
NCT02955355	Phase 3	Immunoglobulin sc Hyqvia	
NCT05084053	Phase 3	Immunoglobulin sc TAK-771	
NCT02027701	Phase 3	Immunoglobulin sc IgPro	
NCT01545076			
NCT04589299	Phase 4	Immunoglobulin sc Hizentra	
NCT04672733			
NCT01757574	Phase 4	Alentuzumab	CD52
Multifocal Motor Neuropathy			
NCT02556437	Phase 2	Immunoglobulin sc Hyqvia	
NCT05225675	Phase 2	ARGX-117	C2
NCT01827072	Phase 3	Immunoglobulin ev NPB-01	
NCT00701662	Phase 3	Immunoglobulin ev vivaglobin	
NCT05084053	Phase 3	Immunoglobulin sc TAK-771	
NCT00666263	Phase 3	Immunoglobulin sc Hyqvia	

## Supplementary Information

Below is the link to the electronic supplementary material.

Supplementary file1 (PDF 3604 kb)

## References

[1] Latov N. Immune mechanisms, the role of complement, and related therapies in autoimmune neuropathies. Expert Rev Clin Immunol. 2021:1–13. 10.1080/1744666X.2021.2002147 34751638

[2] Misawa S, Kuwabara S, Sato Y, Yamaguchi N, Nagashima K, Katayama K (2018). Safety and efficacy of eculizumab in Guillain-Barre syndrome: a multicentre, double-blind, randomised phase 2 trial. Lancet Neurol.

[3] Yamaguchi N, Misawa S, Sato Y, Nagashima K, Katayama K, Sekiguchi Y, et al. A prospective, multicenter, randomized phase II study to evaluate the efficacy and safety of Eeculizumab in patients with Guillain-Barre Syndrome (GBS): Protocol of Japanese Eculizumab Trial for GBS (JET-GBS). JMIR Res Protoc. 2016;5(4):e210. 10.2196/resprot.6610 PMC5118582 27821382

[4] Querol L, Lleixa C. Novel immunological and therapeutic insights in Guillain-Barre syndrome and CIDP. Neurotherapeutics. 2021. 10.1007/s13311-021-01117-3 PMC8455117 34549385

[5] Dalakas MC, Alexopoulos H, Spaeth PJ (2020). Complement in neurological disorders and emerging complement-targeted therapeutics. Nat Rev Neurol.

[6] Roopenian DC, Akilesh S (2007). FcRn: the neonatal Fc receptor comes of age. Nat Rev Immunol.

[7] Lunemann JD, Nimmerjahn F, Dalakas MC (2015). Intravenous immunoglobulin in neurology–mode of action and clinical efficacy. Nat Rev Neurol.

[8] Van den Bergh PYK, van Doorn PA, Hadden RDM, Avau B, Vankrunkelsven P, Allen JA (2021). European Academy of Neurology/Peripheral Nerve Society guideline on diagnosis and treatment of chronic inflammatory demyelinating polyradiculoneuropathy: Report of a joint Task Force-Second revision. J Peripher Nerv Syst.

[9] Briani C, Cocito D, Campagnolo M, Doneddu PE, Nobile-Orazio E. Update on therapy of chronic immune-mediated neuropathies. Neurol Sci. 2021. 10.1007/s10072-020-04998-y 33452933

[10] van Schaik IN, Mielke O, Bril V, van Geloven N, Hartung HP, Lewis RA, et al. Long-term safety and efficacy of subcutaneous immunoglobulin IgPro20 in CIDP: PATH extension study. Neurol Neuroimmunol Neuroinflamm. 2019;6(5):e590. 10.1212/NXI.0000000000000590 PMC6624149 31355323

[11] Cocito D, Grimaldi S, Paolasso I, Falcone Y, Antonini G, Benedetti L, et al. Immunosuppressive treatment in refractory chronic inflammatory demyelinating polyradiculoneuropathy. A nationwide retrospective analysis. Eur J Neurol. 2011;18(12):1417–21. 10.1111/j.1468-1331.2011.03495.x 21819489

[12] Mahdi-Rogers M, Brassington R, Gunn AA, van Doorn PA, Hughes RA. Immunomodulatory treatment other than corticosteroids, immunoglobulin and plasma exchange for chronic inflammatory demyelinating polyradiculoneuropathy. Cochrane Database Syst Rev. 2017;5:CD003280. 10.1002/14651858.CD003280.pub5 PMC6481566 28481421

[13] Briani C, Zara G, Zambello R, Trentin L, Rana M, Zaja F (2004). Rituximab-responsive CIDP. Eur J Neurol.

[14] Benedetti L, Briani C, Franciotta D, Fazio R, Paolasso I, Comi C (2011). Rituximab in patients with chronic inflammatory demyelinating polyradiculoneuropathy: a report of 13 cases and review of the literature. J Neurol Neurosurg Psychiatry.

[15] Roux T, Debs R, Maisonobe T, Lenglet T, Delorme C, Louapre C (2018). Rituximab in chronic inflammatory demyelinating polyradiculoneuropathy with associated diseases. J Peripher Nerv Syst.

[16] Muley SA, Jacobsen B, Parry G, Usman U, Ortega E, Walk D (2020). Rituximab in refractory chronic inflammatory demyelinating polyneuropathy. Muscle Nerve.

[17] Hu J, Sun C, Lu J, Zhao C, Lin J. Efficacy of rituximab treatment in chronic inflammatory demyelinating polyradiculoneuropathy: a systematic review and meta-analysis. J Neurol. 2021. 10.1007/s00415-021-10646-y 34120208

[18] Shimizu S, Iijima M, Fukami Y, Tamura N, Nakatochi M, Ando M, et al. Efficacy and safety of rituximab in refractory CIDP with or without IgG4 autoantibodies (RECIPE): Protocol for a double-blind, randomized, placebo-controlled clinical trial. JMIR Res Protoc. 2020;9(4):e17117. 10.2196/17117 PMC7160709 32234705

[19] Mevorach D, Reiner I, Grau A, Ilan U, Berkun Y, Ta-Shma A, et al. Therapy with eculizumab for patients with CD59 p.Cys89Tyr mutation. Ann Neurol. 2016;80(5):708–17. 10.1002/ana.24770 27568864

[20] Nevo Y, Ben-Zeev B, Tabib A, Straussberg R, Anikster Y, Shorer Z (2013). CD59 deficiency is associated with chronic hemolysis and childhood relapsing immune-mediated polyneuropathy. Blood.

[21] Haliloglu G, Maluenda J, Sayinbatur B, Aumont C, Temucin C, Tavil B (2015). Early-onset chronic axonal neuropathy, strokes, and hemolysis: inherited CD59 deficiency. Neurology.

[22] Vaccaro C, Zhou J, Ober RJ, Ward ES (2005). Engineering the Fc region of immunoglobulin G to modulate in vivo antibody levels. Nat Biotechnol.

[23] Dalakas MC, Spaeth PJ (2021). The importance of FcRn in neuro-immunotherapies: From IgG catabolism, FCGRT gene polymorphisms, IVIg dosing and efficiency to specific FcRn inhibitors. Ther Adv Neurol Disord.

[24] Nelke C, Spatola M, Schroeter CB, Wiendl H, Lunemann JD. Neonatal Fc receptor-targeted therapies in neurology. Neurotherapeutics. 2022. 10.1007/s13311-021-01175-7 PMC9294083 34997443

[25] Takai T (2002). Roles of Fc receptors in autoimmunity. Nat Rev Immunol.

[26] Zhang G, Bogdanova N, Gao T, Song JJ, Cragg MS, Glennie MJ, et al. Fcgamma receptor-mediated inflammation inhibits axon regeneration. PLoS One. 2014;9(2):e88703. 10.1371/journal.pone.0088703 PMC3921223 24523933

[27] He L, Zhang G, Liu W, Gao T, Sheikh KA (2015). Anti-Ganglioside antibodies induce nodal and axonal injury via Fcgamma receptor-mediated inflammation. J Neurosci.

[28] Masuda A, Yoshida M, Shiomi H, Morita Y, Kutsumi H, Inokuchi H (2009). Role of Fc Receptors as a therapeutic target. Inflamm Allergy Drug Targets.

[29] Ben Mkaddem S, Benhamou M, Monteiro RC (2019). Understanding Fc Receptor involvement in inflammatory diseases: From mechanisms to new therapeutic tools. Front Immunol.

[30] Wang X, Mathieu M, Brezski RJ (2018). IgG Fc engineering to modulate antibody effector functions. Protein Cell.

[31] Kang TH, Jung ST (2019). Boosting therapeutic potency of antibodies by taming Fc domain functions. Exp Mol Med.

[32] Uncini A, Mathis S, Vallat JM. New classification of autoimmune neuropathies based on target antigens and involved domains of myelinated fibres. J Neurol Neurosurg Psychiatry. 2021. 10.1136/jnnp-2021-326889 34373238

[33] Querol L, Illa I (2015). Paranodal and other autoantibodies in chronic inflammatory neuropathies. Curr Opin Neurol.

[34] Querol L (2021). Autoimmune nodopathies: treatable neuropathies beyond traditional classifications. J Neurol Neurosurg Psychiatry.

[35] Delmont E, Brodovitch A, Kouton L, Allou T, Beltran S, Brisset M (2020). Antibodies against the node of Ranvier: a real-life evaluation of incidence, clinical features and response to treatment based on a prospective analysis of 1500 sera. J Neurol.

[36] Cortese A, Lombardi R, Briani C, Callegari I, Benedetti L, Manganelli F, et al. Antibodies to neurofascin, contactin-1, and contactin-associated protein 1 in CIDP: Clinical relevance of IgG isotype. Neurol Neuroimmunol Neuroinflamm. 2020;7(1). 10.1212/NXI.0000000000000639 PMC6935837 31753915

[37] Stengel H, Vural A, Brunder AM, Heinius A, Appeltshauser L, Fiebig B, et al. Anti-pan-neurofascin IgG3 as a marker of fulminant autoimmune neuropathy. Neurol Neuroimmunol Neuroinflamm. 2019;6(5). 10.1212/NXI.0000000000000603 PMC6705632 31454780

[38] Fels M, Fisse AL, Schwake C, Motte J, Athanasopoulos D, Gruter T, et al. Report of a fulminant anti-pan-neurofascin-associated neuropathy responsive to rituximab and bortezomib. J Peripher Nerv Syst. 2021. 10.1111/jns.12465 34486194

[39] Pascual-Goni E, Fehmi J, Lleixa C, Martin-Aguilar L, Devaux J, Hoftberger R (2021). Antibodies to the Caspr1/contactin-1 complex in chronic inflammatory demyelinating polyradiculoneuropathy. Brain.

[40] Querol L, Devaux J, Rojas-Garcia R, Illa I (2017). Autoantibodies in chronic inflammatory neuropathies: diagnostic and therapeutic implications. Nat Rev Neurol.

[41] Doppler K, Appeltshauser L, Wilhelmi K, Villmann C, Dib-Hajj SD, Waxman SG (2015). Destruction of paranodal architecture in inflammatory neuropathy with anti-contactin-1 autoantibodies. J Neurol Neurosurg Psychiatry.

[42] Vallat JM, Yuki N, Sekiguchi K, Kokubun N, Oka N, Mathis S (2017). Paranodal lesions in chronic inflammatory demyelinating polyneuropathy associated with anti-Neurofascin 155 antibodies. Neuromuscul Disord.

[43] Koike H, Kadoya M, Kaida KI, Ikeda S, Kawagashira Y, Iijima M (2017). Paranodal dissection in chronic inflammatory demyelinating polyneuropathy with anti-neurofascin-155 and anti-contactin-1 antibodies. J Neurol Neurosurg Psychiatry.

[44] Dalakas MC. IgG4-mediated neurologic autoimmunities: Understanding the pathogenicity of IgG4, ineffectiveness of IVIg, and long-lasting benefits of anti-B cell therapies. Neurol Neuroimmunol Neuroinflamm. 2022;9(1). 10.1212/NXI.0000000000001116 PMC8630661 34845096

[45] Martin-Aguilar L, Lleixa C, Pascual-Goni E, Caballero-Avila M, Martinez-Martinez L, Diaz-Manera J, et al. Clinical and laboratory features in anti-NF155 autoimmune nodopathy. Neurol Neuroimmunol Neuroinflamm. 2022;9(1). 10.1212/NXI.0000000000001098 PMC8564865 34728497

[46] Scheibe F, Ostendorf L, Pruss H, Radbruch H, Aschman T, Hoffmann S, et al. Daratumumab for treatment-refractory antibody-mediated diseases in neurology. Eur J Neurol. 2022. 10.1111/ene.15266 35098616

[47] Beadon K, Guimaraes-Costa R, Leger JM (2018). Multifocal motor neuropathy. Curr Opin Neurol.

[48] Nobile-Orazio E, Giannotta C, Musset L, Messina P, Leger JM (2014). Sensitivity and predictive value of anti-GM1/galactocerebroside IgM antibodies in multifocal motor neuropathy. J Neurol Neurosurg Psychiatry.

[49] Sudo M, Miyaji K, Spath PJ, Morita-Matsumoto K, Yamaguchi Y, Yuki N (2016). Polyclonal IgM and IgA block in vitro complement deposition mediated by anti-ganglioside antibodies in autoimmune neuropathies. Int Immunopharmacol.

[50] Vlam L, Cats EA, Harschnitz O, Jansen MD, Piepers S, Veldink JH, et al. Complement activity is associated with disease severity in multifocal motor neuropathy. Neurol Neuroimmunol Neuroinflamm. 2015;2(4):e119. 10.1212/NXI.0000000000000119 PMC4484896 26161430

[51] Harschnitz O, van den Berg LH, Johansen LE, Jansen MD, Kling S, Vieira de Sa R, et al. Autoantibody pathogenicity in a multifocal motor neuropathy induced pluripotent stem cell-derived model. Ann Neurol. 2016;80(1):71–88. 10.1002/ana.24680 27130524

[52] Budding K, Johansen LE, Van de Walle I, Dijkxhoorn K, de Zeeuw E, Bloemenkamp LM, et al. Anti-C2 antibody ARGX-117 inhibits complement in a disease model for multifocal motor neuropathy. Neurol Neuroimmunol Neuroinflamm. 2022;9(1). 10.1212/NXI.0000000000001107 PMC8587732 34759020

[53] Joint Task Force of the E, the PNS. European Federation of Neurological Societies/Peripheral Nerve Society guideline on management of multifocal motor neuropathy. Report of a joint task force of the European Federation of Neurological Societies and the Peripheral Nerve Society--first revision. J Peripher Nerv Syst. 2010;15(4):295–301. 10.1111/j.1529-8027.2010.00290.x 21199100

[54] Keddie S, Eftimov F, van den Berg LH, Brassington R, de Haan RJ, van Schaik IN. Immunoglobulin for multifocal motor neuropathy. Cochrane Database Syst Rev. 2022;1:CD004429. 10.1002/14651858.CD004429.pub3 PMC8751207 35015296

[55] Harbo T, Andersen H, Jakobsen J (2010). Long-term therapy with high doses of subcutaneous immunoglobulin in multifocal motor neuropathy. Neurology.

[56] Umapathi T, Hughes RA, Nobile-Orazio E, Leger JM. Immunosuppressant and immunomodulatory treatments for multifocal motor neuropathy. Cochrane Database Syst Rev. 2015(3):CD003217. 10.1002/14651858.CD003217.pub5 PMC6781840 25739040

[57] Pestronk A, Florence J, Miller T, Choksi R, Al-Lozi MT, Levine TD (2003). Treatment of IgM antibody associated polyneuropathies using rituximab. J Neurol Neurosurg Psychiatry.

[58] Ruegg SJ, Fuhr P, Steck AJ (2004). Rituximab stabilizes multifocal motor neuropathy increasingly less responsive to IVIg. Neurology.

[59] Stieglbauer K, Topakian R, Hinterberger G, Aichner FT (2009). Beneficial effect of rituximab monotherapy in multifocal motor neuropathy. Neuromuscul Disord.

[60] Chaudhry V, Cornblath DR (2010). An open-label trial of rituximab (Rituxan(R)) in multifocal motor neuropathy. J Peripher Nerv Syst.

[61] Fitzpatrick AM, Mann CA, Barry S, Brennan K, Overell JR, Willison HJ (2011). An open label clinical trial of complement inhibition in multifocal motor neuropathy. J Peripher Nerv Syst.

[62] Latov N, Sherman WH, Nemni R, Galassi G, Shyong JS, Penn AS (1980). Plasma-cell dyscrasia and peripheral neuropathy with a monoclonal antibody to peripheral-nerve myelin. N Engl J Med.

[63] Campagnolo M, Ruiz M, Falzone YM, Ermani M, Bianco M, Martinelli D (2019). Limitations in daily activities and general perception of quality of life: Long term follow-up in patients with anti-myelin-glycoprotein antibody polyneuropathy. J Peripher Nerv Syst.

[64] Delmont E, Hiew FL, Cassereau J, Aube-Nathier AC, Grapperon AM, Attarian S (2017). Determinants of health-related quality of life in anti-MAG neuropathy: a cross-sectional multicentre European study. J Peripher Nerv Syst.

[65] Nobile-Orazio E, Meucci N, Baldini L, Di Troia A, Scarlato G (2000). Long-term prognosis of neuropathy associated with anti-MAG IgM M-proteins and its relationship to immune therapies. Brain.

[66] Lunn MP, Nobile-Orazio E. Immunotherapy for IgM anti-myelin-associated glycoprotein paraprotein-associated peripheral neuropathies. Cochrane Database Syst Rev. 2016;10:CD002827. 10.1002/14651858.CD002827.pub4 PMC6457998 27701752

[67] Dalakas MC, Rakocevic G, Salajegheh M, Dambrosia JM, Hahn AF, Raju R (2009). Placebo-controlled trial of rituximab in IgM anti-myelin-associated glycoprotein antibody demyelinating neuropathy. Ann Neurol.

[68] Leger JM, Viala K, Nicolas G, Creange A, Vallat JM, Pouget J (2013). Placebo-controlled trial of rituximab in IgM anti-myelin-associated glycoprotein neuropathy. Neurology.

[69] Hughes R, Bensa S, Willison H, Van den Bergh P, Comi G, Illa I (2001). Randomized controlled trial of intravenous immunoglobulin versus oral prednisolone in chronic inflammatory demyelinating polyradiculoneuropathy. Ann Neurol.

[70] Campagnolo M, Zambello R, Nobile-Orazio E, Benedetti L, Marfia GA, Riva N (2017). IgM MGUS and Waldenstrom-associated anti-MAG neuropathies display similar response to rituximab therapy. J Neurol Neurosurg Psychiatry.

[71] Svahn J, Petiot P, Antoine JC, Vial C, Delmont E, Viala K (2018). Anti-MAG antibodies in 202 patients: clinicopathological and therapeutic features. J Neurol Neurosurg Psychiatry.

[72] Gazzola S, Delmont E, Franques J, Boucraut J, Salort-Campana E, Verschueren A (2017). Predictive factors of efficacy of rituximab in patients with anti-MAG neuropathy. J Neurol Sci.

[73] Dalakas MC (2017). Rituximab in anti-MAG neuropathy: More evidence for efficacy and more predictive factors. J Neurol Sci.

[74] Broglio L, Lauria G (2005). Worsening after rituximab treatment in anti-mag neuropathy. Muscle Nerve.

[75] Gironi M, Saresella M, Ceresa L, Calvo M, Ferrante P, Merli F, et al. Clinical and immunological worsening in a patient affected with Waldenstrom macroglobulinemia and anti-mag neuropathy after treatment with rituximab. Haematologica. 2006;91(6 Suppl):ECR17. 16785123

[76] Noronha V, Fynan TM, Duffy T. Flare in neuropathy following rituximab therapy for Waldenstrom's macroglobulinemia. J Clin Oncol. 2006;24(1):e3. 10.1200/JCO.2005.04.6474 16382111

[77] Sala E, Robert-Varvat F, Paul S, Camdessanche JP, Antoine JC (2014). Acute neurological worsening after Rituximab treatment in patients with anti-MAG neuropathy. J Neurol Sci.

[78] Castillo JJ, Advani RH, Branagan AR, Buske C, Dimopoulos MA, D'Sa S (2020). Consensus treatment recommendations from the tenth International Workshop for Waldenstrom Macroglobulinaemia. Lancet Haematol.

[79] Vo ML, Martin P, Latov N (2015). Correlation of Changes in Gait Parameters, With Phenotype, Outcome Measures, and Electrodiagnostic Abnormalities in a Patient With Anti-MAG Neuropathy After Exacerbation and Improvement. J Clin Neuromuscul Dis.

[80] Paul F, Cartron G (2019). Infusion-related reactions to rituximab: frequency, mechanisms and predictors. Expert Rev Clin Immunol.

[81] Massa F, Zuppa A, Pesce G, Demichelis C, Bergamaschi M, Garnero M, et al. Bendamustine-rituximab (BR) combined therapy for treatment of immuno-mediated neuropathies associated with hematologic malignancy. J Neurol Sci. 2020;413:116777. 10.1016/j.jns.2020.116777 32200107

[82] Latov N (2014). Diagnosis and treatment of chronic acquired demyelinating polyneuropathies. Nat Rev Neurol.

[83] Morabito F, Tripepi G, Del Poeta G, Mauro FR, Reda G, Sportoletti P (2021). Effectiveness of ibrutinib as first-line therapy for chronic lymphocytic leukemia patients and indirect comparison with rituximab-bendamustine: Results of study on 486 cases outside clinical trials. Am J Hematol.

[84] Visentin A, Frezzato F, Severin F, Imbergamo S, Pravato S, Romano Gargarella L (2020). Lights and shade of next-generation Pi3k inhibitors in chronic lymphocytic leukemia. Onco Targets Ther.

[85] Visentin A, Imbergamo S, Gurrieri C, Frezzato F, Trimarco V, Martini V (2017). Major infections, secondary cancers and autoimmune diseases occur in different clinical subsets of chronic lymphocytic leukaemia patients. Eur J Cancer.

[86] Furman RR, Eradat HA, DiRienzo CG, Hofmeister CC, Hayman SR, Leonard JP (2017). Once-weekly ofatumumab in untreated or relapsed Waldenstrom's macroglobulinaemia: an open-label, single-arm, phase 2 study. Lancet Haematol.

[87] Hauser SL, Bar-Or A, Cohen JA, Comi G, Correale J, Coyle PK (2020). Ofatumumab versus Teriflunomide in Multiple Sclerosis. N Engl J Med.

[88] Goede V, Klein C, Stilgenbauer S (2015). Obinutuzumab (GA101) for the treatment of chronic lymphocytic leukemia and other B-cell non-hodgkin's lymphomas: a glycoengineered type II CD20 antibody. Oncol Res Treat.

[89] Mossner E, Brunker P, Moser S, Puntener U, Schmidt C, Herter S (2010). Increasing the efficacy of CD20 antibody therapy through the engineering of a new type II anti-CD20 antibody with enhanced direct and immune effector cell-mediated B-cell cytotoxicity. Blood.

[90] Suresh T, Lee LX, Joshi J, Barta SK (2014). New antibody approaches to lymphoma therapy. J Hematol Oncol.

[91] Rakocevic G, Martinez-Outschoorn U, Dalakas MC. Obinutuzumab, a potent anti-B-cell agent, for rituximab-unresponsive IgM anti-MAG neuropathy. Neurol Neuroimmunol Neuroinflamm. 2018;5(4):e460. 10.1212/NXI.0000000000000460 PMC5886834 29629397

[92] Briani C, Visentin A, Salvalaggio A, Cacciavillani M, Trentin L (2019). Obinutuzumab, a new anti-CD20 antibody, and chlorambucil are active and effective in anti-myelin-associated glycoprotein antibody polyneuropathy. Eur J Neurol.

[93] Briani C, Visentin A, Cerri F, Quattrini A (2020). From pathogenesis to personalized treatments of neuropathies in hematological malignancies. J Peripher Nerv Syst.

[94] Treon SP, Tripsas CK, Meid K, Warren D, Varma G, Green R (2015). Ibrutinib in previously treated Waldenstrom's macroglobulinemia. N Engl J Med.

[95] Dimopoulos MA, Trotman J, Tedeschi A, Matous JV, Macdonald D, Tam C (2017). Ibrutinib for patients with rituximab-refractory Waldenstrom's macroglobulinaemia (iNNOVATE): an open-label substudy of an international, multicentre, phase 3 trial. Lancet Oncol.

[96] Vos JM, Notermans NC, D'Sa S, Lunn MP, van der Pol WL, Kraan W (2018). High prevalence of the MYD88 L265P mutation in IgM anti-MAG paraprotein-associated peripheral neuropathy. J Neurol Neurosurg Psychiatry.

[97] Castellani F, Visentin A, Campagnolo M, Salvalaggio A, Cacciavillani M, Candiotto C, et al. The Bruton tyrosine kinase inhibitor ibrutinib improves anti-MAG antibody polyneuropathy. Neurol Neuroimmunol Neuroinflamm. 2020;7(4). 10.1212/NXI.0000000000000720 PMC7176252 32284437

[98] Owen RG, McCarthy H, Rule S, D'Sa S, Thomas SK, Tournilhac O (2020). Acalabrutinib monotherapy in patients with Waldenstrom macroglobulinemia: a single-arm, multicentre, phase 2 study. Lancet Haematol.

[99] Tam CS, Opat S, D'Sa S, Jurczak W, Lee HP, Cull G (2020). A randomized phase 3 trial of zanubrutinib vs ibrutinib in symptomatic Waldenstrom macroglobulinemia: the ASPEN study. Blood.

[100] Tam CS, Dimopoulos MA, Garcia-Sanz R, Trotman J, Opat S, Roberts AW, et al. Pooled safety analysis of zanubrutinib monotherapy in patients with B-cell malignancies. Blood Adv. 2021. 10.1182/bloodadvances.2021005621 PMC8864647 34724705

[101] Dimopoulos M, Sanz RG, Lee HP, Trneny M, Varettoni M, Opat S (2020). Zanubrutinib for the treatment of MYD88 wild-type Waldenstrom macroglobulinemia: a substudy of the phase 3 ASPEN trial. Blood Adv.

[102] Kater AP, Wu JQ, Kipps T, Eichhorst B, Hillmen P, D'Rozario J (2020). Venetoclax Plus Rituximab in Relapsed Chronic Lymphocytic Leukemia: 4-Year Results and Evaluation of Impact of Genomic Complexity and Gene Mutations From the MURANO Phase III Study. J Clin Oncol.

[103] Castillo JJ, Allan JN, Siddiqi T, Advani RH, Meid K, Leventoff C, et al. Venetoclax in previously treated Waldenstrom Macroglobulinemia. J Clin Oncol. 2021:JCO2101194. 10.1200/JCO.21.01194 PMC8683218 34793256

[104] Monaco S, Bonetti B, Ferrari S, Moretto G, Nardelli E, Tedesco F (1990). Complement-mediated demyelination in patients with IgM monoclonal gammopathy and polyneuropathy. N Engl J Med.

[105] Briani C, Ferrari S, Campagnolo M, Tagliapietra M, Castellani F, Salvalaggio A, et al. Mechanisms of nerve damage in neuropathies associated with hematological diseases: Lesson from nerve biopsies. Brain Sci. 2021;11(2). 10.3390/brainsci11020132 PMC7909400 33498362

[106] Amaador K, Wieske L, Koel-Simmelink MJA, Kamp A, Jongerius I, de Heer K, et al. Serum neurofilament light chain, contactin-1 and complement activation in anti-MAG IgM paraprotein-related peripheral neuropathy. J Neurol. 2022. 10.1007/s00415-022-10993-4 PMC9217848 35157138

[107] Doets AY, Hughes RA, Brassington R, Hadden RD, Pritchard J. Pharmacological treatment other than corticosteroids, intravenous immunoglobulin and plasma exchange for Guillain-Barre syndrome. Cochrane Database Syst Rev. 2020;1:CD008630. 10.1002/14651858.CD008630.pub5 PMC6984651 31981368

[108] Ruck T, Nimmerjahn F, Wiendl H, Lunemann JD. Next generation antibody-based therapies in neurology. Brain. 2021. 10.1093/brain/awab465 PMC9630709 34928330

[109] Mastellos DC, Ricklin D, Lambris JD (2019). Clinical promise of next-generation complement therapeutics. Nat Rev Drug Discov.

[110] Roghanian A, Teige I, Martensson L, Cox KL, Kovacek M, Ljungars A (2015). Antagonistic human FcgammaRIIB (CD32B) antibodies have anti-tumor activity and overcome resistance to antibody therapy in vivo. Cancer Cell.

